# A 10 year retrospective review of factors associated with poor foetal outcome in patients with placenta praevia at the University of Maiduguri Teaching Hospital, Nigeria

**DOI:** 10.4314/ahs.v24i2.25

**Published:** 2024-06

**Authors:** Ado D Geidam, Hassan H Abubakar

**Affiliations:** 1 Department of Obstetrics and Gynaecology, College of Medical Sciences, University of Maiduguri; 2 Department of Obstetrics and Gynaecology, University of Maiduguri Teaching Hospital, Maiduguri

**Keywords:** Foetal outcome, placenta praevia, poor outcome, UMTH

## Abstract

**Background:**

Placenta Praevia (PP), a placenta that is implanted in the lower uterine segment has the potential to cause severe obstetric complications including foetal death.

**Objective:**

To determine the factors associated with poor foetal outcomes in patients with placenta praevia.

**Method:**

A retrospective review of the foetal outcome of all cases of PP managed at the University of Maiduguri Teaching Hospital over 10 years (2011 to 2021).

Chi-square test or Fixer exact test as appropriate were used to determine the factors associated with the development of poor foetal outcome. P-value < 0.05 was consider statistically significant.

**Results:**

There were 26,407 deliveries during the study period out of which 166 were placenta previa; a prevalence of 0.6%. Most of the patients, 84.8% (95/112) were unbooked. The majority 50.9% (57/112) had blood loss ≥ 1000 ml and 30.4% (34/112) foetuses were of low birth weight. Low birthweight, hypotension, anaemia, unbooked status, vaginal delivery, and EBL ≥ 1000 mls were found to be significantly associated with foetal death.

**Conclusions:**

Poor foetal outcome was associated with Unbooked status, anaemia, vaginal delivery, EBL of ≥ 1000 mls, hypotension and duration of hospital stay ≥ 7 days in patients with placenta previa in our environment.

## Introduction

Placenta Praevia (PP) which describes a placenta that is implanted in the lower uterine segment, either over or very near (within 0.1–2.0 cm) the internal cervical os is an obstetrics condition that has the potential to cause severe obstetric complications including foetal death.^1^,^2^ This is because bleeding occurs when the cervix dilates and/or effaces during labour causing the placenta to separate from the underlying decidua.^3^ Bleeding can also occur before labour with formation and effacement of the lower uterine segment with advancing gestation as well as prelabour uterine contraction.^4^

Placenta praevia occurs in 2.8/1000 singleton pregnancies and 3.9/1000 twin pregnancies,^5^,^6^ and a prevalence of 0.8%^7^ and 1.65%^8^ have been reported in Nigeria.

The maternal risk associated with PP includes blood transfusion, postpartum anaemia, hysterectomy, septicemia, thrombophlebitis and maternal death.^9^ Neonates born after pregnancies complicated by placenta previa have a higher risk of being born preterm, being of low birth weight, having a low Apgar score, requiring intensive neonatal care, being stillbirth or dying in the neonatal period.^10^,^11^ Also, the rate of foetal abnormality in patients with PP doubles the background rate of the general population and overall 10 to 15% of women with bleeding placenta previa have a coexistent abruption which is also associated with foetal death.^12^

Knowing the factors associated with poor foetal outcome (death) can help in devising means to prevent it. For example, the identified causes can be closely followed up and judiciously managed to prevent the development of a poor foetal outcome. Therefore, this study aimed to determine the factors associated with poor foetal outcomes in patients with placenta previa in our environment. It is hoped that the findings of this study may aid in formulating measures that will improve foetal outcomes of placenta previa in our environment.

## Method

This was a retrospective review of the foetal outcome of all cases of placenta praevia managed at the University of Maiduguri Teaching Hospital over 10 years (2011 to 2021).

The patient's identification data were retrieved from the labour, antenatal, postnatal and gynaecology emergency wards, admission and discharge record books and the operation theatre's register. The identification data was used to retrieve the case notes of the patients from the Medical Records department.

Information on socio-demographic characteristics, parity, booking status, gestational age at diagnosis, clinical presentation, predisposing factors, pre and postoperative PCV, mode of diagnosis, type of praevia, gestational age at delivery, mode of delivery, estimated blood loss, management, complications, duration of hospital stay, and foetal outcomes were collected using a proforma designed for the study.

Data collected were analyzed using Statistical Package for Social Sciences (SPSS) version 25 (IBM SPSS Statistic). All data were presented as absolute values and percentages in tables and figures. Chi-square test or Fixer exact test were used to determine the factors associated with the development of poor foetal outcome (foetal death). Odd ratio and 95% confidence interval were also determined. P-value < 0.05 was considered statistically significant.

Ethical clearance was obtained from the UMTH ethical and research committee.

## Results

There were 26,407 deliveries during the period under review. Of these, 166 patients had placenta praevia giving a prevalence of 0.6%. Of these 166 cases, only 148 files were retrieved of which 112 case files had complete information for a retrieval rate of 89%.

Most of the patients were less than 35 years of age 67% (75/112). Many of the patients 59.8% (67/112) were of less than 5 parity and most 84.8% (95/112) were unbooked. The majority of the patients 89.3% (100/112) presented with vaginal bleeding. Only 10.7% (12/112) were asymptomatic and were diagnosed incidentally during an ultrasound scan. In 92.7% (104/112) of the cases, the placenta praevia was major. More than half of the cases 81.2% (91/112) were delivered as soon as they presented after initial resuscitation through caesarean section while 18.8% (21/112) of patients were managed expectantly. Most of the patients 62.5% (70/112) had diastolic BP of > 60mmHg and 97.3% (109/112) had systolic BP of > 90mmHg.

One hundred and four patients underwent caesarean section (92.8%), of which 81.2% (91/112)) were emergency caesarean section. Nine of the patients had PPH (8%) while two of the patients were admitted to ICU and a caesarean hysterectomy was done in one of the patients who had morbidly adherent placenta. There were sixteen (14.3%) foetal deaths. About 14.3% (16/112) of the babies had birth asphyxia while 30.4% (34/112) were of low birth weight and 52.7% (59/112) were delivered preterm. About half 50.9% (57/112) had blood loss of 1000 ml or and fourteen patients 12.6% stayed more than 10 days on admission.

Identifiable factors associated with foetal death in this study include low birthweight, hypotension, anaemia, unbooked status, vaginal delivery, EBL of ≥ 1000 mls and duration of hospital stay ≥ 7 days. (Table 3)

**Table 3:** Factors associated with poor neonatal outcome (neonatal death) in the patients with PP

Characteristics	Alive	Death	χ^2^ (P-value)	Odd Ratio (95%CI)
**Diagnosis**				
GA at Delivery				
<37 weeks	48 (81.4%)	11(18.6%)	1.934 (0.164)	0.727(0.494 – 1.070)
≥37 weeks	48 (90.6%)	5(9.4%)		
**Birth Weight**				
<2.5Kg	25(73.5%)	9(26.5%)	5.920(0.015)	2.950(1.197 – 7.268)
≥2.5Kg	71(91.0%)	7(9.0%)		
**Systolic BP**				
<90mmHg	1(33.3%)	2(66.7%)	6.907(0.009)	1.668(1.190 – 2.339)
≥90mmHg	95(87.2)	14(12.8)		
**Duration of hospital stay**				
≥7days	21(70.0%)	9(30.0%)	8.263(0.004)	4.592(1.529 – 13.793)
<7days	75(91.5%)	7(8.5%)		
**Parity group**				
<5	58(86.6%)	9(13.4%)	0.099(0.753)	1.187(0.408 – 3.458)
≥5	38(84.4%)	7(15.6%)		
**PCV on admission**				
<30	35(70.0%)	15(30.0%)	18.215(<0.001)	10.167(1.515 – 68.230)
≥30	61(98.4)	1(1.6%)		
**Diastolic BP**				
<60mmHg	29(69.0%)	13(31.0%)	15.244(<0.001)	3.722(1.331 – 10.410)
≥60mmHg	67(95.7%)	3(4.3%)		
**Estimated Blood Loss**				
≥1000mls	43(75.4%)	14(24.6%)	10.009(0.002)	4.417(1.193 – 16.350)
<1000mls	53(96.4%)	2(3.6%)		
**Booking status**				
Booked	17(100.0%)	0(0.0%)	3.340(0.068)	0.823(0.750 – 0.903)
Unbooked	79(83.25)	16(16.8%)		
**Age group**				
<35 years	66(88.0%)	9(12.0%)	0.969(0.325)	1.711(0.582 – 5.028)
≥35 years	30(81.1%)	7(18.9%)		
**Fetal sex**				
Male	41(85.4%)	7(14.6%)	0.006(0.938)	0.958(0.330 – 2.787)
Female	55(85.9%)	9(14.1%)		
**Type of PP**				
Major	88(84.6%)	16(15.4%)	1.436(0.231)	0.328(0.042 – 2.562)
Minor	8(100.0%)	0(0.0%)		
**Diagnosis/Presentation**				
Asymptomatic	12(100.0%)	0(0.0%)	2.240(0.134)	1.639(0.559 – 4.799)
PV bleeding	84(84.0%)	16(16.0%)		
**Mode of Delivery**				
Vaginal	2(25.0%)	6(75.0%)	25.936(<0.001)	28.200(5.009 – 158.749)
CS	94(90.4%)	10(9.6%		

## Discussion

This study showed low birthweight, hypotension, anaemia, unbooked status, vaginal delivery, EBL of ≥ 1000 mls and duration of hospital stay ≥ 7 days were significantly associated with neonatal death in patients with placenta praevia.

Low birth weight was found to be associated with 3 folds increased risk of neonatal death in this study. Similar findings were also reported by Schneiderman et al. and Awad et al.^13^,^14^ However, Sameh Mashaly et al. and Afzal et al 4,15 did not find any significant association between birth weight and neonatal outcome. Birth weight is an important prognostic factor for foetal health as it reflects the nutritional and metabolic conditions of the mother, as well as foetal development during pregnancy. Also, the lower the birth weight and the gestational age, the greater the chance of foetal death^16^ and in this study majority of the deliveries 52.7% were preterm. Sub analysis revealed that the LBW babies were born preterm.

Placenta praevia can result in life-threatening haemorrhage and this study found a significant association between EBL > 1000mls and hypotension/shock with adverse foetal outcomes as systolic BP of < 90 mmHg and diastolic BP < 60 mmHg were found to have about 2 folds and about 4 folds risk of poor foetal outcome. Similar findings were also reported elsewhere.^16^,^17^,^18^,^19^

In this study, there was a statistical association between anaemia on admission and foetal death and it was found to increase the risk of foetal death by about 10folds. A similar finding was reported by Lone et al. 20 Maternal anaemia increases the risk for preterm delivery, low birth weight and stillbirth as a result of decreased oxygen and nutrient delivery to the placenta.

The significant association between duration of hospital stay ≥ 7 and poor foetal outcome in this study could be an effect rather than a cause as those people with anaemia, LBW and EBL >1000mls (found to be significantly associated with a poor foetal outcome) may/span> require longer admission for adequate treatment.

Antenatal care can play an important part in the improvement of foeto-maternal outcome in PP as it allows early diagnosis of PP, timely arrangement of blood, admiration of steroids and involvement of a multidisciplinary team. In this study, most of the patients were unbooked. Similar findings were reported elsewhere.^19^,^21^,^22^ There was also an association between being booked and fetal outcome. Being booked is associated with reduced risk of foetal death with the OR of 0.823 (CI=0.750-0.903). A similar finding was reported by Tayyiba Wasim et al.^23^

Our study found a significant association between adverse neonatal outcomes and mode of delivery. Those that were delivered vaginally have 28 times increased risk of poor foetal outcome than those delivered via caesarean section. Vaginal delivery may entail a delay compared to caesarean delivery and this is detrimental with ongoing vaginal bleeding.

## Conclusion

Unbooked status, anaemia, vaginal delivery, EBL of ≥ 1000 mls and hypotension were associated with poor foetal outcomes. Thus, good antenatal care including more frequent antenatal check-ups, correction of anaemia during the antenatal period, anticipating the complications, and educating patients regarding the complications is recommended to improve foetal outcome with placenta praevia.

## Figures and Tables

**Table 1:** Shows the socio-demographic and clinical characteristics of the study population

Characteristics	Frequency	Percentages
**Age group**		
<35	75	67.0
≥35	37	33.0
Total	112	100.0
**Parity**		
<5	67	59.8
≥5	45	40.2
Total	112	100.0
**Booking Status**		
Unbooked	95	84.8
Booked	17	15.2
Total	112	100.0
**Clinical Presentation**		
Asymptomatic	12	10.7
PV Bleeding	100	89.3
Total	112	100.0
**Type of PP**		
Minor	8	7.1
Major	104	92.9
Total	112	100.0
**Management**		
Expectant	21	18.8
C/S	91	81.3
Total	112	100.0
**Diastolic BP**		
≤60	42	37.5
>60	70	62.5
Total	112	100.0
**Systolic BP**		
<90	3	2.7
≥90	109	97.3
Total	112	100.0
**PCV on admission**		
<30	50	44.6
≥30	62	55.4
Total	112	100.0

**Table 2:** Pregnancy outcome of the study group

Outcome	Frequency	Percentage
**Mode of Delivery**		
Vaginal	8	7.1
CS	104	92.9
Total	112	100.0
**PPH**		
Yes	9	8.0
No	103	92.0
Total	112	100.0
**Hysterectomy**		
Yes	1	0.9
No	111	99.1
Total	112	100.0
**ICU admission**		
Yes	2	1.8
No	110	98.2
Total	112	100.0
**Foetal outcome**		
Alive	96	85.7
Death	16	14.3
Total	112	100.0
**Foetal sex**		
Male	48	42.9
Female	64	57.1
Total	112	100.0
**Apgar 5**		
≥7	96	85.7
<7	16	14.3
Total	112	100.0
**Estimated Blood Loss (EBL)**		
≥1000	57	50.9
<1000	55	49.1
Total	112	100.0
**Duration of hospital stay**		
>7days	30	26.8
≤7days	82	73.2
Total	112	100.0
**Birth Weight**		
<2.5kg	34	30.4
≥2.5kg	78	69.6
Total	112	100.0
**GA delivery**		
<37 weeks	59	52.7
≥37 weeks	53	47.3

## Data Availability

The datasets generated and analysed during the current study are available from the corresponding author on reasonable request.
